# Protection of the ischaemic heart: investigations into the phenomenon of ischaemic preconditioning

**Published:** 2009-02

**Authors:** A Lochner, E Marais, S Genade, B Huisamen, EF Du Toit, JA Moolman

**Affiliations:** Department of Biomedical Sciences (Section Medical Physiology), Faculty of Health Sciences, University of Stellenbosch, Stellenbosch; Department of Biomedical Sciences (Section Medical Physiology), Faculty of Health Sciences, University of Stellenbosch, Stellenbosch; Department of Biomedical Sciences (Section Medical Physiology), Faculty of Health Sciences, University of Stellenbosch, Stellenbosch; Department of Biomedical Sciences (Section Medical Physiology), Faculty of Health Sciences, University of Stellenbosch, Stellenbosch; Department of Biomedical Sciences (Section Medical Physiology), Faculty of Health Sciences, University of Stellenbosch, Stellenbosch; Department of Biomedical Sciences (Section Medical Physiology), Faculty of Health Sciences, University of Stellenbosch, Stellenbosch

## Abstract

**Summary:**

Exposure of the heart to one or more short episodes of ischaemia/reperfusion protects the heart against a subsequent prolonged period of ischaemia, as evidenced by a reduction in infarct size and an improvement in functional recovery during reperfusion. Elucidation of the mechanism of this endogenous protection could lead to the development of pharmacological mimetics to be used in the clinical setting. The aim of our studies was therefore to gain more information regarding the mechanism of ischaemic preconditioning, using the isolated perfused working rat heart as model.

A preconditioning protocol of 1 × 5 or 3 × 5 min of ischaemia, interspersed with 5 min of reperfusion was found to protect hearts exposed to 25 min of global ischaemia or 35–45 min of regional ischaemia. These models were used throughout our studies.

In view of the release of catecholamines by ischaemic tissue, our first aim was to evaluate the role of the alphaadrenergic receptor in ischaemic preconditioning. However, using a multi-cycle ischaemic preconditioning protocol, we could not find any evidence for alpha-1 adrenergic or PKC activation in the mechanism of preconditioning. Cyclic increases in the tissue cyclic nucleotides, cAMP and cGMP were found, however, to occur during a multi-cycle preconditioning protocol, suggesting roles for the beta-adrenergic signalling pathway and nitric oxide (NO) as triggers of cardioprotection. This was substantiated by the findings that (1) administration of the beta-adrenergic agonist, isoproterenol, or the NO donors SNAP or SNP before sustained ischaemia also elicited cardioprotection similar to ischaemic preconditioning; (2) beta-adrenergic blockade or nitric oxide synthase inhibition during an ischaemic preconditioning protocol abolished protection. Effectors downstream of cAMP, such as p38MAPK and CREB, were also demonstrated to be involved in the triggering process.

Our next step was to evaluate intracellular signalling during sustained ischaemia and reperfusion. Our results showed that ischaemic preconditioned-induced cardioprotection was associated with a significant reduction in tissue cAMP, attenuation of p38MAPK activation and increased tissue cGMP levels and HSP27 activation, compared to non-preconditioned hearts. The role of the stress kinase p38MAPK was further investigated by using the inhibitor SB203580. Our results suggested that injury by necrosis and apoptosis share activation of p38MAPK as a common signal transduction pathway and that pharmacological targeting of this kinase offers a tenable option to manipulate both these processes during ischaemia/reperfusion injury.

## Summary

Cardiovascular disease remains a leading cause of morbidity and mortality in the western world and according to the predictions of the World Health Organisation, it will be the major cause of death worldwide by the year 2020.^1^ There is therefore continued interest in developing new drugs and interventions that will limit the extent of infarction and prevent cell death.

The discovery by Murry and co-workers in 1986,^2^ that exposure of the heart to four cycles of 5-min ischaemia, interspersed with 5 min of reperfusion significantly reduced infarct size, indicated that the heart has a significant endogenous protective mechanism at its disposal. This phenomenon, termed ischaemic preconditioning, has been recognised as the ‘strongest form of *in vivo* protection against myocardial ischaemic injury other than early reperfusion’.^3^ This, in turn, has led to an enormous effort to elucidate the mechanism of preconditioning, the rationale being that should the mechanism of protection be known, it could lead to the development of pharmacological mimetics to be used in the clinical setting. The interest that this phenomenon has evoked is also reflected in the large number of reviews that have appeared on the topic.^4^-^6^ This phenomenon was subsequently found to occur in all species and organs tested (for a review see reference 4).

In 1991, our laboratory decided to join the race for the discovery of the mechanism of ischaemic preconditioning. Our first task was to develop and characterise a model of ischaemic preconditioning, using the isolated, perfused, working rat heart as experimental model. Using functional recovery during reperfusion as the endpoint, we found that subjecting hearts to a preconditioning protocol of one episode of 5 min of global ischaemia, followed by 5 min of reperfusion, before a sustained global ischaemic period of 25 min, caused a significant improvement in post-ischaemic function and structural appearance.^7^

However, controversial results obtained by workers using different animal species, experimental protocols, models and endpoints prompted us to re-evaluate the role of the model (retrograde vs working heart perfusion), endpoints (functional recovery vs infarct size) and degree of ischaemia (global vs regional ischaemia), respectively, in the outcome of prior preconditioning. The results obtained showed that while the working, preconditioned rat heart (1 × 5 min ischaemia) showed a significant improvement in post-ischaemic functional recovery, it was more difficult to demonstrate improved function in the retrogradely perfused heart. Preconditioning of working hearts showed a significant decline in infarct size after both 30 and 35 min regional ischaemia, while the retrogradely perfused heart showed a significant decline after 35 min only. The results also indicated that infarct size was a more reliable endpoint than functional recovery.^8^ In fact, most workers in the field currently use infarct size as the gold standard for evaluation of cardio-protection induced by prior preconditioning.

## Characterisation of events during an ischaemic preconditioning protocol

To gain more information regarding the mechanism of preconditioning, we argued that knowledge of events during an ischaemic preconditioning protocol is a prerequisite for identifying the mechanisms involved. It is now well established that three endogenous triggers are released during exposure of the heart to short episodes of ischaemia/reperfusion, namely adenosine, opioids and bradykinin (for a review see references 9, 10). Their respective roles in eliciting protection have been demonstrated using appropriate receptor agonists and antagonists, which were able to elicit or abolish cardioprotection, respectively.

The role of the release of endogenous catecholamines in eliciting preconditioning received surprisingly little attention. Ischaemia-mediated release of catecholamines and a concomitant increase in tissue cAMP have been known for many years,^11^ and in the initial studies on preconditioning, a role for the α_1_-adrenergic receptor was proposed.^12^

Therefore, our first attempt to elucidate the mechanism of ischaemic preconditioning was aimed at establishing the involvement of the α_1_-adrenergic signalling pathway and its downstream substrate PKC. Using a preconditioning protocol of 3 × 5 min global ischaemia in the working rat heart model, we tested the effectiveness of preconditioning in the presence of the α_1_-adrenergic blocker, prazosin, and the selective PKC blockers, chelerythrine and bisindolylmaleimide, as well as the ability of repetitive α_1_-adrenergic activation using phenylephrine to mimic preconditioning. In this model, we could not find any evidence for α_1_-adrenergic or PKC activation in the mechanism of preconditioning.^13^ In retrospect, it is possible that the multicycle preconditioning protocol (3 × 5 min) used in these experiments was the reason for these negative results because it is now known that such a preconditioning protocol elicits a powerful form of protection, which is more difficult to inhibit than a single episode.^4^

**Fig. 1.**
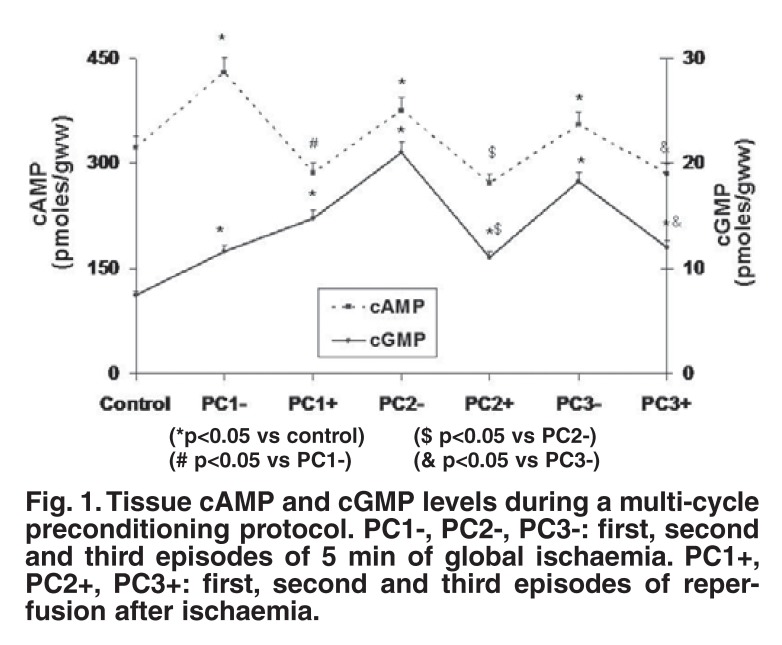
Tissue cAMP and cGMP levels during a multi-cycle preconditioning protocol. PC1-, PC2-, PC3-: first, second and third episodes of 5 min of global ischaemia. PC1+, PC2+, PC3+: first, second and third episodes of reperfusion after ischaemia.

The observation that the cyclic nucleotides cAMP and cGMP increased in a cyclic fashion at the end of each preconditioning episode suggested roles for the beta-adrenergic signal transduction system as well as NO^14^-^16^ as triggers in the preconditioning process (Fig. 1).

**Fig. 2.**
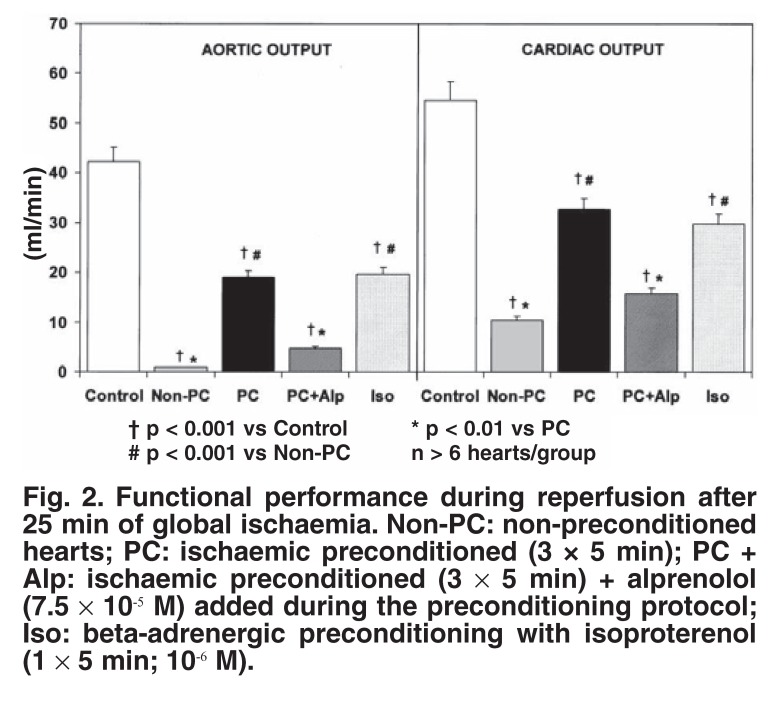
Functional performance during reperfusion after 25 min of global ischaemia. Non-PC: non-preconditioned hearts; PC: ischaemic preconditioned (3 × 5 min); PC + Alp: ischaemic preconditioned (3 × 5 min) + alprenolol (7.5 × 10^-5^ M) added during the preconditioning protocol; Iso: beta-adrenergic preconditioning with isoproterenol (1 × 5 min; 10^-6^ M).

**Fig. 3.**
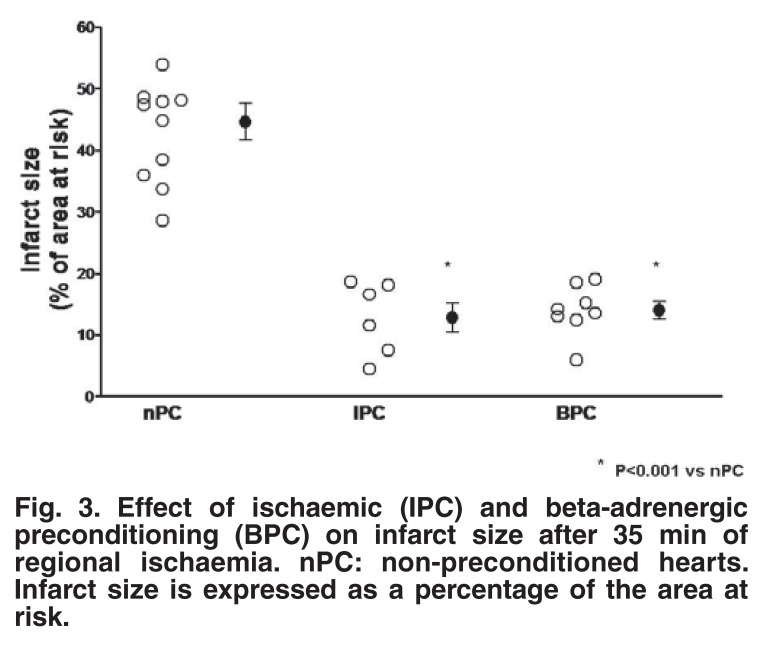
Effect of ischaemic (IPC) and beta-adrenergic preconditioning (BPC) on infarct size after 35 min of regional ischaemia. nPC: non-preconditioned hearts. Infarct size is expressed as a percentage of the area at risk.

The contribution of β-adrenergic receptor stimulation to triggering preconditioning was proven by the use of appropriate agonists and antagonists: administration of the β_1_-adrenergic blocker, alprenolol (7.5 × 10^-5^ M) during the triggering phase significantly attenuated (but did not abolish) cardioprotection, 15 whereas pharmacological activation of the β-adrenergic receptor (1 × 5 min administration of 10^-6^–10^-8^ M isoproterenol) caused a significant improvement in functional recovery during reperfusion,^15^ as well as a reduction in infarct size^17^ (Figs 2, 3). Pharmacological preconditioning with β-adrenergic activation (the so-called beta-adrenergic preconditioning) has also been demonstrated by Nasa *et al.*,^18^ Asimakis and Conti^19^ and Robinet *et al.*^20^

**Fig. 4.**
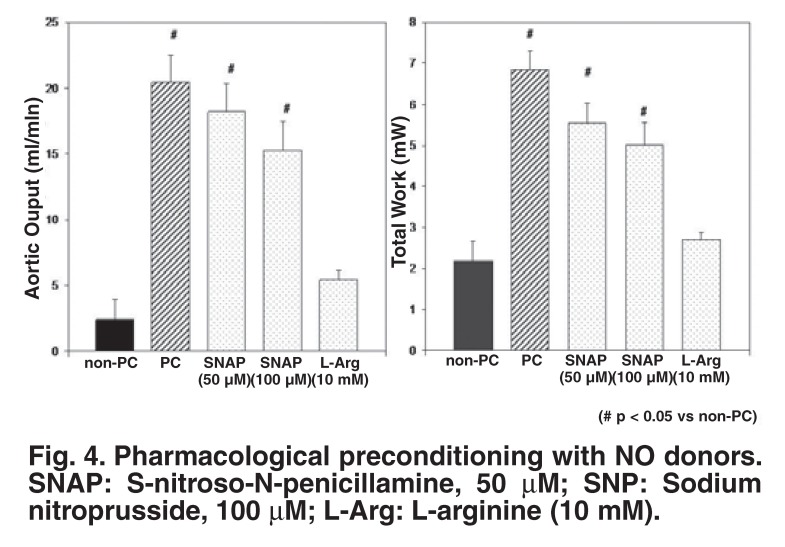
Pharmacological preconditioning with NO donors. SNAP: S-nitroso-N-penicillamine, 50 μM; SNP: Sodium nitroprusside, 100 μM; L-Arg: L-arginine (10 mM).

**Fig. 5.**
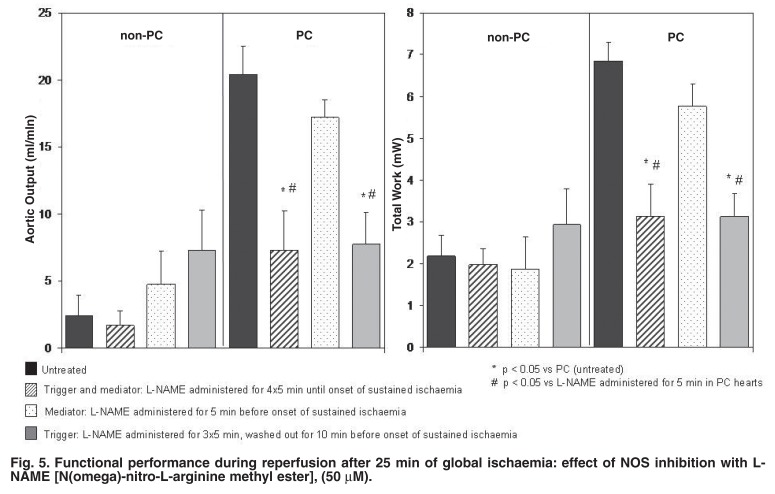
Functional performance during reperfusion after 25 min of global ischaemia: effect of NOS inhibition with LNAME [N(omega)-nitro-L-arginine methyl ester], (50 μM).

The role of the cyclic increases in cGMP in eliciting cardioprotection was subsequently investigated, using a similar experimental approach to that above.^14^ Tissue cyclic nucleotides were manipulated using NO donors [e.g. S-nitroso-N-penicillamine (SNAP) and sodiumnitroprusside (SNP)] and inhibitors of nitric oxide synthase (NOS) (e.g. L-NAME or LNA). Pharmacological elevation in tissue cGMP levels by SNAP or SNP before sustained ischaemia improved post-ischaemic functional recovery comparable to that of ischaemic preconditioning (Fig. 4), while administration of the NOS inhibitors before and during the preconditioning protocol attenuated functional recovery (Fig. 5).

Later studies confirmed that exogenous NO was able to elicit preconditioning-induced protection.^4^ Subsequent studies also emphasised the significance of cGMP and activation of PKG in the triggering of preconditioning. In 2005, Costa *et al.*^21^ showed that addition of exogenous PKG and cGMP to isolated mitochondria resulted in the opening of the mitochondrial K_ATP_ channels, via PKCε. This, in turn, leads to the generation of reactive oxygen species (ROS), which acts as a second messenger in triggering cardioprotection.^22^,^23^

## Signalling events during triggering of preconditioning

Having established the involvement of the second messengers, cAMP and cGMP in triggering ischaemic and pharmacological preconditioning, we investigated the downstream signalling events, focusing on the mitogen-activated protein kinase (MAPKinase) family. Each subfamily of the MAPK family, ERK, JNK and p38 MAPK, has been suggested to play a role in the cardioprotection elicited by prior preconditioning (for reviews see references 24, 25). In view of the many controversial results obtained, we decided initially to focus on the role of p38 MAPK as a trigger by evaluating its activation pattern during a multi-cycle ischaemic preconditioning protocol and during β-adrenergic preconditioning.

**Fig. 6.**
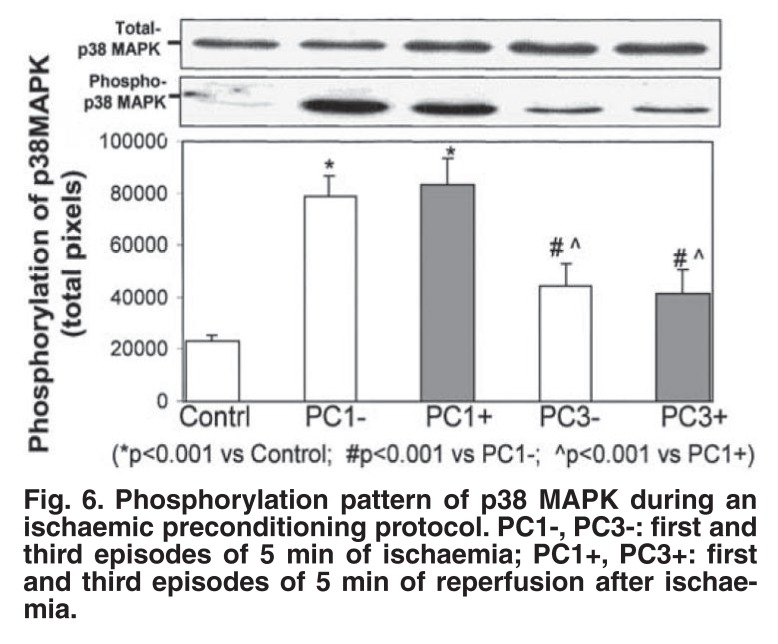
Phosphorylation pattern of p38 MAPK during an ischaemic preconditioning protocol. PC1-, PC3-: first and third episodes of 5 min of ischaemia; PC1+, PC3+: first and third episodes of 5 min of reperfusion after ischaemia.

The results showed that although p38 MAPK is significantly activated during the first and second ischaemic preconditioning episodes, the activation is transient and disappears after the third episode (Fig. 6).^26^ Beta-adrenergic preconditioning with isoproterenol also caused a significant dose-dependent activation of the kinase within three minutes. These cyclic elevations in p38 MAPK during an ischaemic preconditioning protocol are most likely due to the release of substances acting as triggers, for example endogenous catecholamines. The transient nature of p38 MAPK activation does not argue against its putative role as trigger of protection, since its downstream effects may persist long after its initial activation.^26^

The triggering action of p38 MAPK activation in both ischaemic and β-adrenergic preconditioning was further substantiated by the finding that β-adrenergic blockade with alprenolol during the preconditioning protocol inhibited p38 MAPK activation, and inhibition of its activation by SB 203580 in β-adrenergic preconditioning abolished cardioprotection. However, a number of observations argued against p38 MAPK activation as the only trigger in a multi-cycle preconditioning protocol, since bracketing this protocol with SB203580 did not abolish protection. 26 Schneider and co-workers,^27^ using the p38MAPK inhibitor SB202190, also failed to block preconditioning-induced improvement in contractile recovery and reduction in infarct size in an isolated rat heart model.

However, we have subsequently shown that SB203580 abolishes cardioprotection elicited by a single-episode preconditioning protocol, which indicates that the other triggers known to be involved in a multi-cycle protocol may override the triggering actions of p38 MAPK.^28^ The triggering role of p38 MAPK was further substantiated by using anisomycin, an activator of p38 MAPK,^28^ which could also elicit cardioprotection, as evidenced by a reduction in infarct size.

At this stage, the sequence of events downstream of p38 MAPK still needed further investigation. MAPKAPK-2 and -3 (mitogen-activated protein kinase-activated protein kinase 2 and 3) and PRAK (p38-regulated and activated kinase), located downstream of p38 MAPK^29^,^30^ are known to phosphorylate the 27 kDa small heat-shock protein (HSP27), which has been shown to protect against ischaemic stress.^31^ We therefore evaluated the role of HSP27 as downstream effector of p38 MAPK during an ischaemic or β-adrenergic preconditioning protocol.^32^

**Fig. 7.**
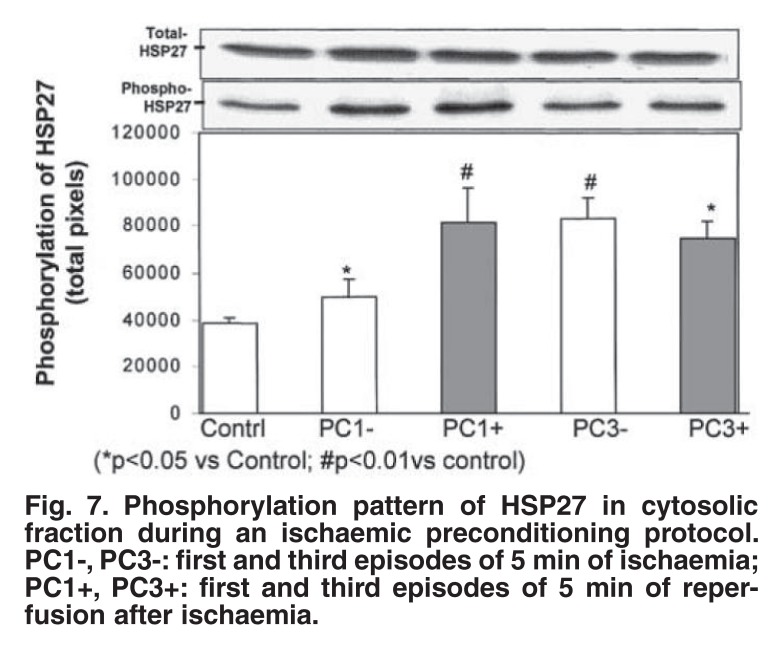
Phosphorylation pattern of HS P27 in cytosolic fraction during an ischaemic preconditioning protocol. PC1-, PC3-: first and third episodes of 5 min of ischaemia; PC1+, PC3+: first and third episodes of 5 min of reperfusion after ischaemia.

Interestingly, the marked, but transient activation of p38 MAPK during a multi-cycle ischaemic preconditioning protocol was associated with sustained activation of HSP27 throughout this procedure (Fig. 7). Similar changes in p38 MAPK and HSP27 occurred during β-adrenergic preconditioning. Pretreatment with SB 203580 abolished activation of both p38 MAPK and HSP27, suggesting that p38 MAPK activation triggers HSP27 phosphorylation. Both these events appear to be important in eliciting cardioprotection, since inhibition with SB203580 abolished protection, as indicated by a reduction in functional recovery and an increased infarct size.^32^

**Fig. 8.**
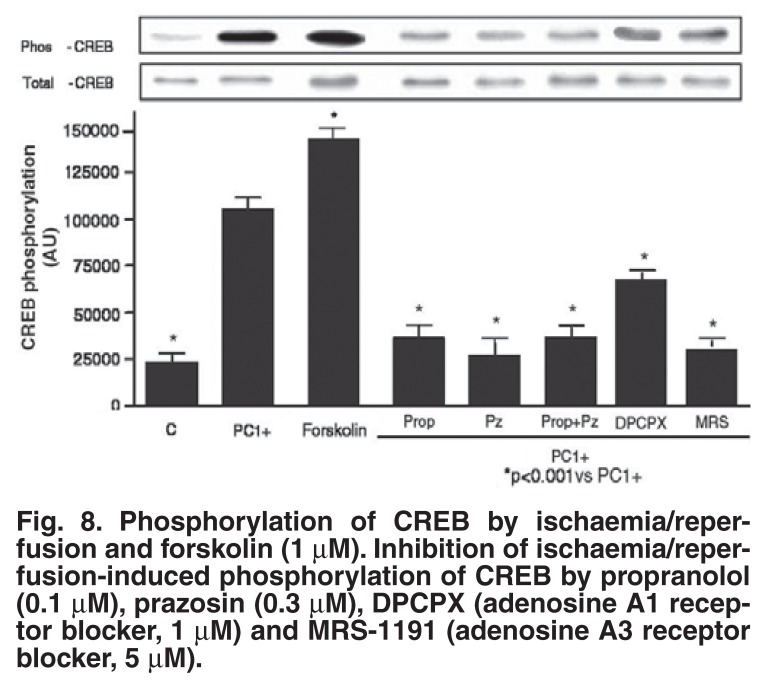
Phosphorylation of CREB by ischaemia/reperfusion and forskolin (1 μM). Inhibition of ischaemia/reperfusion-induced phosphorylation of CREB by propranolol (0.1 μM), prazosin (0.3 μM), DPCPX (adenosine A1 receptor blocker, 1 μM) and MRS-1191 (adenosine A3 receptor blocker, 5 μM).

**Fig. 9.**
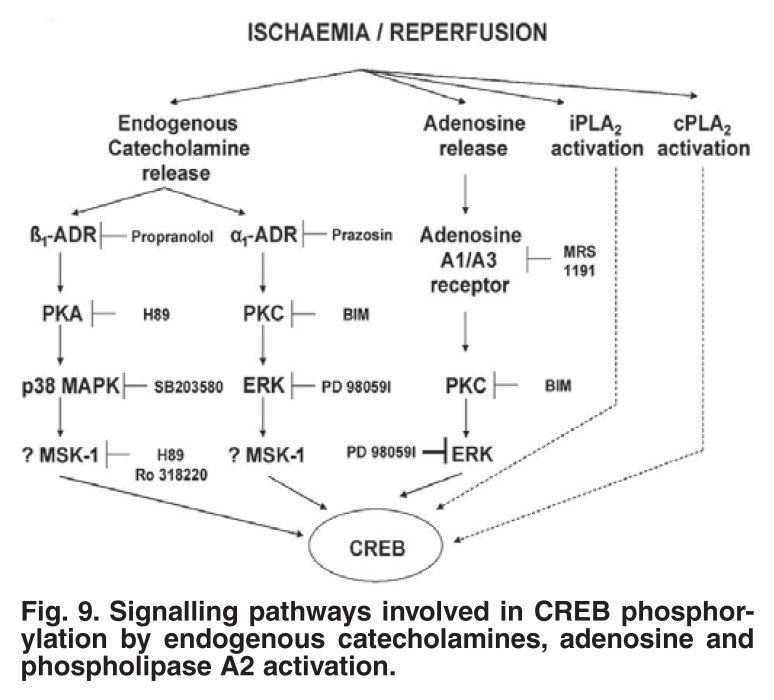
Signalling pathways involved in CREB phosphorylation by endogenous catecholamines, adenosine and phospholipase A2 activation.

Finally, the role of the transcription factor, cyclic AMP response element-binding protein (CREB), as possible downstream effector of p38 MAPK was evaluated during an ischaemic preconditioning protocol.^33^ As in the case of HSP27, CREB was activated by exposure of the heart to 5 min of ischaemia, followed by reperfusion, and it remained activated throughout the multi-cycle ischaemic preconditioning protocol. Release of endogenous catecholamines activates CREB via both the α_1_- and β_1_-adrenergic receptors, while the adenosine A_1_ and A_3_ receptors are also involved, as indicated by the use of selective antagonists (Fig. 8). CREB activation by events downstream of receptor stimulation included activation of PKA, PKC, ERK, MSK-1 and p38 MAPK. These observations indicate that CREB may well be a convergence point for several signalling pathways during the triggering process of preconditioning^33^ (Fig. 9).

Interestingly, both the Ca^2+^-independent (iPLA2) and cytosolic (cPLA_2_) phospholipases are involved in CREB activation during ischaemia/reperfusion. This was demonstrated by the use of the inhibitors 4-bromo-enol-lactone and AACOF_3_, respectively.^33^ These phospholipases also play a significant role in cardioprotection, since administration of these inhibitors during a singlecycle preconditioning protocol significantly increased infarct size. As far as we know, this was the first demonstration of the involvement of these phospholipases in ischaemic preconditioning, and their significance in this scenario needs to be further investigated.

Finally, our data confirm the crucial role of p38 MAPK and its downstream targets, for example, HSP27 and CREB in triggering the cardioprotection of both ischaemic and β-adrenergic preconditioning. A recent study by Nagy *et al.*^34^ indicated that MSK-1 is an alternative (other than MAPKAPK-2 and HSP27) downstream target for p38 MAPK, which then transmits the survival signal through activation of CREB. In view of the above, it is possible that gene expression was activated during a multicycle preconditioning protocol. However, events downstream of CREB phosphorylation and their link(s) to cardioprotection during sustained ischaemia and particularly during reperfusion still remain to be established.

## Signalling events during sustained ischaemia and reperfusion

**Cyclic nucleotides**

After having established that the ischaemic preconditioning process can be triggered by, among others, β-adrenergic stimulation as well as NO and a number of the downstream events in this process, we characterised events during sustained ischaemia in an attempt to gain more information regarding the effectors of protection.

Our initial study focused on the possibility that prior preconditioning protects via energy sparing during sustained ischaemia. Careful monitoring of tissue high-energy phosphate and glycogen levels, as well as lactate accumulation suggested that prior preconditioning reduced energy utilisation during sustained ischaemia in preconditioned hearts.^7^ Similar energy-sparing effects were reported by others.^35^,^36^ Whether these changes were sufficient to account for the improvement in recovery or whether they are merely the consequence of protection elicited by preconditioning, needs to be further investigated.

**Fig. 10.**
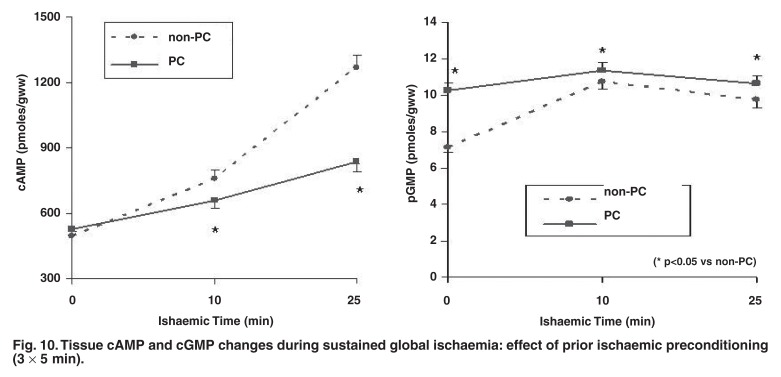
Tissue cAMP and cGMP changes during sustained global ischaemia: effect of prior ischaemic preconditioning (3 × 5 min).

In view of the role of β-adrenergic stimulation as trigger, our next step was to evaluate this particular signalling pathway during sustained ischaemia. The deleterious consequences of activation of the β-adrenergic signal transduction pathway in myocardial ischaemia are widely appreciated. Indeed, our results showed that cAMP accumulation during sustained ischaemia was significantly less in preconditioned hearts,^37^ and associated with upregulation of cAMP- and cGMP-phosphodiesterase activities.^16^ (Fig. 10).

Reduced cAMP accumulation during sustained ischaemia was also observed in preconditioned rabbit hearts.^38^,^39^ The question was then raised whether the reduction in cAMP was the cause or result of preconditioning-induced protection. Our approach was to evaluate the characteristics of the different members of the β-adrenergic signal transduction pathway at the onset of sustained ischaemia (i.e. immediately after ischaemic preconditioning). The B_max_ of the β-receptor was found to be increased by 39% and its Kd decreased by 35%. Adenylyl cyclase and PKA activations were reduced at this stage and desensitised to further β-adrenergic stimulation.^15^ Similar reduced responsiveness to β-adrenergic signal transduction in preconditioned hearts was reported by Simonis *et al.*^40^

To assess whether a reduced tissue cAMP content *per se* is related to cardioprotection, tissue cAMP was elevated experimentally by administration of forskolin (which directly activates adenylyl cyclase) to preconditioned hearts. Although it markedly increased tissue cAMP levels during sustained ischaemia, it did not abolish protection.^37^ Sandhu and co-workers^38^ used NKH477 to activate adenylyl cyclase during ischaemia and likewise found no loss of protection in rabbit hearts. These data strongly suggested that the reduced cAMP levels during sustained ischaemia in preconditioned hearts were probably merely a reflection of protection, rather than a causal factor.

In contrast to cAMP, a significant increase in tissue cGMP during sustained ischaemia was observed in preconditioned hearts.^14^ The significance of NOS and GMP as triggers in classic preconditioning was discussed in the previous section. However, little is known about the role of cGMP during sustained ischaemia of the preconditioned heart. It is known to reduce intracellular Ca^2+^ and cause vasorelaxation.^41^ It has also been shown that exogenous PKG and cGMP, when added to isolated mitochondria, resulted in opening of the mitochondrial K_ATP_ channels. 21 Whether this contributes to protection of the heart during sustained ischaemia as well remains to be established.

## p38MAPK

**Fig. 11.**
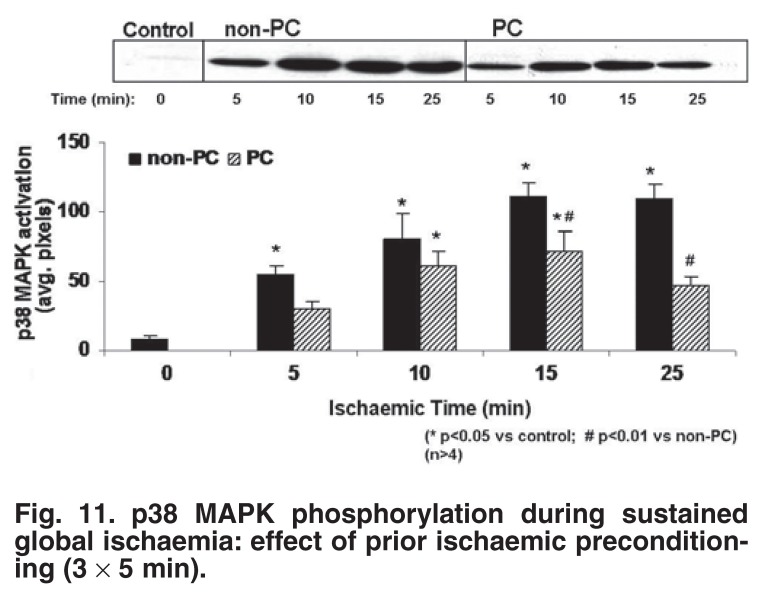
p38 MAPK phosphorylation during sustained global ischaemia: effect of prior ischaemic preconditioning (3 × 5 min).

**Fig. 12.**
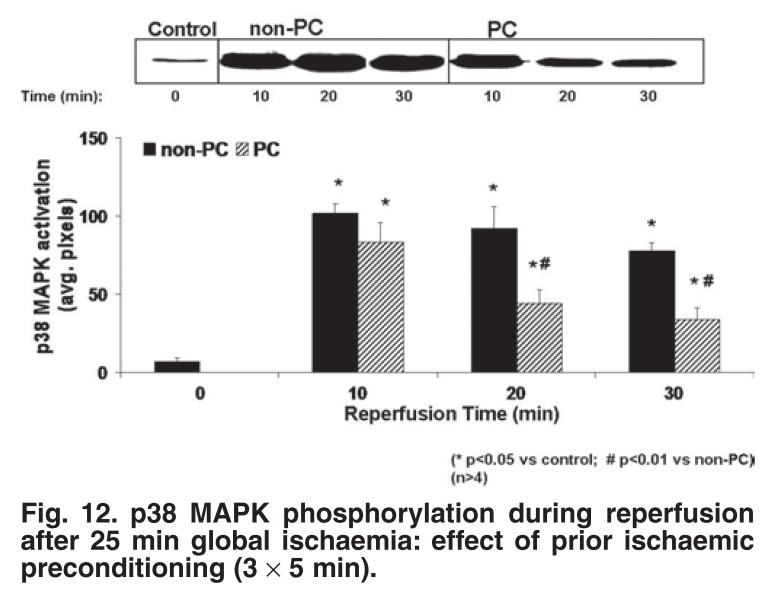
p38 MAPK phosphorylation during reperfusion after 25 min global ischaemia: effect of prior ischaemic preconditioning (3 × 5 min).

The role of p38 MAPK activation during sustained ischaemia and reperfusion was the next to be addressed.^26^,^28^,^32^ It was known that myocardial ischaemia/reperfusion activates p38 MAPK in most species,^42^-^44^ with the exception of the rabbit heart.^45^,^46^ In our study, dual phosphorylation and activation of p38 MAPK were monitored at 5, 10, 15 and 25 min of sustained global ischaemia of non-preconditioned and ischaemic preconditioned hearts. p38MAPK was significantly less in preconditioned than in non-preconditioned hearts during both ischaemia and reperfusion (Figs 11, 12).^26^ This pattern was also observed in β-adrenergic preconditioning, or hearts preconditioned with the NO donors SNAP and SNP.^47^ Conversely, inhibition of preconditioning during the triggering phase by β-blockade, p38 MAPK inhibition or NOS inhibition, resulted in an increase in p38 MAPK activation during sustained ischaemia, similar to that observed in non-preconditioned hearts.

In summary, in our hands, attenuation of p38 MAPK activation during sustained ischaemia and reperfusion was associated with improved mechanical recovery during reperfusion and a reduction in infarct size.^17^,^26^ Conversely, maintained high levels of p38 MAPK activation during ischaemia/reperfusion were present in hearts that failed to recover mechanically and failed to show a reduction in infarct size.^17^

Significantly less p38 MAPK activation has also been reported in preconditioned rat myoblasts^29^ and perfused hearts,^48^ as well as dog hearts *in vivo*.^44^ Inhibition of p38 MAPK during or immediately before sustained ischaemia, by administration of the inhibitors SB203580 or SB202190, is also cardioprotective.^49^,^50^ Perhaps the most convincing evidence in this regard came from a study on neonatal myocytes.^51^ Not only did SB203580 reduce ischaemic injury, but prior preconditioning of these cells prevented p38α MAPK activation during ischaemia. Moreover, cells expressing a dominant negative p38α, which prevented p38 MAPK activation, were resistant to lethal ischaemia.

Despite these convincing data, other workers published contradictory findings, suggesting an association between increased activation of p38MAPK and cardioprotection.^45^,^52^ It would appear that the outcome of p38 MAPK activation in ischaemia is dependent on the isoform activated and it is possible that the p38 MAPK isoforms are differentially activated by ischaemic preconditioning. It has been demonstrated that transgenic mice over-expressing p38α-MAPKdn were significantly protected from myocardial ischaemia/reperfusion injury.^53^ Determining the precise role of the p38 MAPK pathway in ischaemic damage will ultimately rely on the development of p38 MAPK isoform-selective inhibitors and new p38 MAPK-targeting agents.^54^

However, despite conflicting results reported regarding p38 MAPK activation in cardioprotection, our results obtained in the perfused rat heart provide strong evidence of a detrimental role of p38 MAPK activation in the setting of ischaemia/reperfusion. Our subsequent studies were therefore aimed at determining how attenuation of p38MAPK activation confers protection against ischaemia/reperfusion damage.

## How does attenuation of p38 MAPK activation protect the heart against ischaemic damage?

Ischaemic preconditioning protects the myocardium against the various deleterious effects of ischaemia, such as necrosis^2^ and apoptosis.^55^,^56^ It is well established that necrosis occurs during ischaemia as well as during early reperfusion, while apoptosis occurs mainly during reperfusion,^57^,^58^ the latter making an independent contribution to reperfusion injury. It has been suggested that the stress kinases may activate important signal transduction pathways in apoptosis, caused by ischaemia/reperfusion.^59^ For example, Ma and co-workers^60^ showed that activation of p38 MAPK during myocardial ischaemia/reperfusion caused apoptosis.

Since the role of attenuation of p38 MAPK activation in protection against apoptosis in ischaemic preconditioning was not known at that stage, we investigated whether the reduction in apoptosis known to occur in ischaemic preconditioning was the result of inhibition of p38 MAPK during ischaemia/reperfusion and whether β-adrenergic preconditioning also protected against apoptosis.

We also attempted to establish whether activation of p38 MAPK during the preconditioning protocol (trigger phase) was involved in the anti-apoptotic effect of both ischaemic and β-adrenergic preconditioning.^17^ In this study, apoptosis was measured by caspase-3 activation and PARP cleavage.

Our results showed that attenuation of p38 MAPK activation during sustained ischaemia of both ischaemic and β-adrenergic preconditioned hearts was associated with a reduction in apoptosis as well as infarct size. These results suggested that injury by necrosis and apoptosis share activation of p38 MAPK as a common signal transduction pathway and that pharmacological targeting of p38 MAPK offers a tenable option to manipulate both apoptosis and necrosis during ischaemia/reperfusion injury. Interestingly, in contrast to the many investigations focusing on the role of the kinases (p38MAPK in particular), very little indeed is known about the role of the phosphatases in this scenario.

**Fig. 13.**
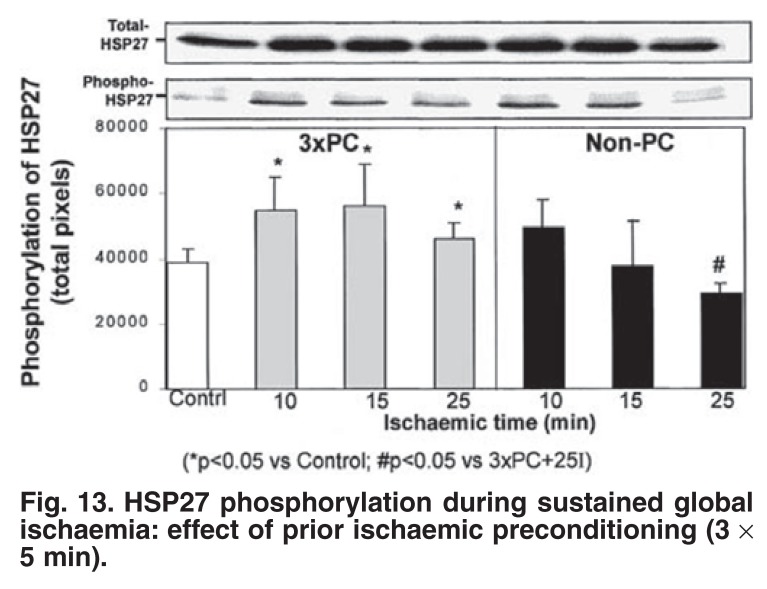
HS P27 phosphorylation during sustained global ischaemia: effect of prior ischaemic preconditioning (3 × 5 min).

Co-existence of attenuated p38 MAPK activation and cardioprotection does not indicate causality and the question remains, as in the case of cAMP levels in ischaemia, whether attenuation of this stress kinase is merely a reflection of the protected myocardium or whether it causes protection. In a study on the temporal relationship between p38 MAPK and HSP27, we found that attenuation of p38 MAPK during sustained ischaemia of ischaemic preconditioned and β-adrenergic preconditioned hearts was associated with phosphorylation of both cytosolic and myofibrillar HSP27 (Fig. 13).^32^

The cardioprotective actions of the small heat-shock proteins are well-established.^31^,^61^,^62^ For example, over-expression of HSP27 in cultured cardiac cells has a potent cardioprotective effect,^62^,^63^ and mice over-expressing HSP 27 were protected from lethal ischaemia/reperfusion injury.^64^ HSPs may confer protection in several ways, for example they may act as chaperones,^65^ or stabilise the cytoskeleton66 or actin to accelerate recovery from stress.^67^,^68^ A recent study reported a novel role for HSP27 in cardioprotection: it protected cardiac troponin I and troponin T from ischaemia/reperfusion-induced degradation by preventing their proteolytic clearage via interaction with these proteins. Such protection resulted in restored post-ischaemic myofilament response to Ca^2+^ and improved contractile function.^69^ These data suggest that increased activation of the small heat-shock proteins during sustained ischaemia could indeed be involved in preconditioning-induced cardioprotection.

## Conclusions

Our work thus far on the mechanism of ischaemic preconditioning has identified beta-adrenergic stimulation as an important trigger in the process. Careful elucidation of downstream signalling indicated activation of the stress kinase p38 MAPK as a trigger, while attenuation of its activation during sustained ischaemia and reperfusion was associated with a reduction in necrosis and apoptosis. Experimental manipulation of p38 MAPK activation suggested a possible causal role in triggering cardioprotection. How the attenuated activation of this stress kinase during ischaemia and reperfusion is linked to cardioprotection remains to be established.

Recently, attention has shifted to events during early reperfusion where activation of the so-called RISK pathway (ERK and PKB/Akt) and inhibition of the formation of the mitochondrial permeability transition pore have been suggested to be the final effector of cardioprotection.^5^,^6^,^9^ Since it is believed that the kinases are merely signalling molecules that carry the protective signal, it remains to be established whether and how p38MAPK is linked to these final effectors in cardioprotection.
